# Mortality, incidence, and microbiological documentation of ventilated acquired pneumonia (VAP) in critically ill patients with COVID-19 or influenza

**DOI:** 10.1186/s13613-023-01207-9

**Published:** 2023-10-30

**Authors:** Guillaume Laurichesse, Carole Schwebel, Niccolò Buetti, Mathilde Neuville, Shidasp Siami, Yves Cohen, Virginie Laurent, Bruno Mourvillier, Jean Reignier, Dany Goldgran‐Toledano, Stéphane Ruckly, Etienne de Montmollin, Bertrand Souweine, Jean‐François Timsit, Claire Dupuis, for the OUTCOME R. E. A. network

**Affiliations:** 1 Pneumology and infectious diseases Gabriel montpied hospital, Clermont Ferrand University Hospital, 63000 Clermont Ferrand, France; 2 Medical Intensive Care Unit, University Hospital, Grenoble‐Alpes, 38000 Grenoble, France; 3 UMR 1137, IAME, Université Paris Cité, 75018 Paris, France; 4 Infection Control Program and WHO Collaborating Centre on Patient Safety, Faculty of Medicine, University of Geneva Hospitals, 1205 Geneva, Switzerland; 5 Polyvalent Intensive Care Unit, Hôpital Foch, 92150 Suresnes, France; 6 General Intensive Care Unit, Sud Essonne Hospital, 91150 Etampes, France; 7 Intensive Care Unit, University Hospital Avicenne, AP‐HP, 93000 Bobigny, France; 8 Polyvalent Intensive Care Unit, André Mignot Hospital, 78150 Le Chesnay, France; 9 Medical Intensive Care Unit, University Hospital of Reims, 51100 Reims, France; 10 Medical Intensive Care Unit, University Hospital of Nantes, 44000 Nantes, France; 11 Medical and Surgical Intensive Care, Montfermeil Hospital, 93370 Montfermeil, France; 12 Medical and Infectious Diseases Intensive Care Unit, Bichat Hospital, AP‐HP, Paris Cité University, 46rue Henri Huchard, 75018 Paris, France; 13 Medical Intensive Care Unit, University Hospital Gabriel Montpied, 63000 Clermont‐Ferrand, France; 14 Université Clermont Auvergne, UMR CNRS 6023 LMGE, 63000 Clermont-Ferrand, France; 15 Unité de Nutrition Humaine, CRNH Auvergne, INRAe, Université Clermont Auvergne, 63000 Clermont Ferrand, France

## Abstract

**Background:**

Data on ventilator associated pneumonia (VAP) in COVID-19 and influenza patients admitted to intensive care units (ICU) are scarce. This study aimed to estimate day-60 mortality related to VAP in ICU patients ventilated for at least 48 h, either for COVID-19 or for influenza, and to describe the epidemiological characteristics in each group of VAP.

**Design:**

Multicentre retrospective observational study.

**Setting:**

Eleven ICUs of the French OutcomeRea^™^ network.

**Patients:**

Patients treated with invasive mechanical ventilation (IMV) for at least 48 h for either COVID-19 or for flu.

**Results:**

Of the 585 patients included, 503 had COVID-19 and 82 had influenza between January 2008 and June 2021. A total of 232 patients, 209 (41.6%) with COVID-19 and 23 (28%) with influenza, developed 375 VAP episodes. Among the COVID-19 and flu patients, VAP incidences for the first VAP episode were, respectively, 99.2 and 56.4 per 1000 IMV days (*p* < 0.01), and incidences for all VAP episodes were 32.8 and 17.8 per 1000 IMV days (*p* < 0.01). Microorganisms of VAP were Gram-positive cocci in 29.6% and 23.5% of episodes of VAP (*p *< 0.01), respectively, including *Staphylococcus aureus* in 19.9% and 11.8% (*p* = 0.25), and Gram-negative bacilli in 84.2% and 79.4% (*p* = 0.47). In the overall cohort, VAP was associated with an increased risk of day-60 mortality (aHR = 1.77 [1.36; 2.30], *p* < 0.01), and COVID-19 had a higher mortality risk than influenza (aHR = 2.22 [CI 95%, 1.34; 3.66], p < 0.01). VAP was associated with increased day-60 mortality among COVID-19 patients (aHR = 1.75 [CI 95%, 1.32; 2.33], *p* < 0.01), but not among influenza patients (aHR = 1.75 [CI 95%, 0.48; 6.33], *p* = 0.35).

**Conclusion:**

The incidence of VAP was higher in patients ventilated for at least 48 h for COVID-19 than for influenza. In both groups, Gram-negative bacilli were the most frequently detected microorganisms. In patients ventilated for either COVID-19 or influenza VAP and COVID-19 were associated with a higher risk of mortality.

**Supplementary Information:**

The online version contains supplementary material available at 10.1186/s13613-023-01207-9.

## Background

Since January 2020, the SARS-CoV2 infection has caused acute respiratory failure resulting in numerous patients requiring intensive care unit (ICU) admission and invasive mechanical ventilation (IMV) [1]. Several studies have assessed the outcome of ICU patients admitted for COVID-19 and those admitted with other respiratory viral illnesses. Among other viruses that can cause pandemics influenza plays an important role in terms of morbidity and mortality [2].

Most viral infectious diseases weaken local or systemic immunity and often lead to other infectious complications as described in influenza with pneumococcal or staphylococcal pneumonia [3]. Nosocomial infections and particularly ventilator-associated pneumonia (VAP) are one of the leading causes of deaths in the ICU setting [4].

There are differences between COVID-19 and influenza in respiratory pathophysiology, clinical course and patient management, such as the primary cellular targets and the cell binding receptors on the virus surface, the duration of incubation and clinical course prior to ICU admission, the severity of lower respiratory tract inflammation, and the use of corticosteroids or other anti-inflammatory treatments. Thus, the risk for and the consequences of VAP in critically ill patients requiring mechanical ventilation for acute respiratory failure may vary widely in both COVID-19 and influenza.

Several studies have reported data on the epidemiology and consequences of bacterial VAP in patients with COVID-19 [5–7] or H1N1 influenza [8], most of which concerned risk factors, length of hospital stay and mortality [5–7]. Few comparisons were made between the two viruses regarding the occurrence of VAP. VAP was more frequently observed in COVID-19 than in influenza or in other infections, or in patients with no infection, but the diagnostic procedures may have differed between these settings and bacterial identification may have been less frequently performed in COVID-19 for fear of contamination [9].

The main objective of the present study was to assess the association between VAP and the risk of day-60 mortality in ICU patients ventilated for at least 48 h for SARS-CoV-2 pneumonia and with influenza pneumonia. The secondary objectives were to estimate the incidence of VAP in each group and to characterize the etiologic microorganisms and outcome of VAP.

## Materials and methods

### Data source

We carried out a retrospective analysis from the French perspective multicentre OutcomeRea^™^ database (*n* = 11 ICUs). The methods of data collection and quality of the database have been described in detail elsewhere [10, 11].

### Ethical considerations

In accordance with French law, the OutcomeRea™ database was approved by the Advisory Committee for Data Processing in Health Research (CCTIRS) and the National Commission for Data Protection and Liberties (CNIL, Registration No. 8999262). The database protocol was submitted to the Institutional Review Board of the Clermont-Ferrand University Hospital which waived the need for informed consent (IRB No. 5891).

### Study population

Patients over 18 years admitted to one of the ICUs belonging to the OutcomeRea™ network between January 2008 and June 2021 were included if they had laboratory-confirmed SARS-CoV-2 or influenza pneumonia and had been treated with IMV for at least 48 h.

Patients were excluded if they had been referred from another ICU when a decision was made to discontinue life-sustaining treatments during the first two calendar days after ICU admission, if they had both COVID-19 and influenza pneumonia, and if they had been ICU discharged after June 2021.

### Data collection

All data were prospectively collected including demographics, chronic disease/comorbidities as assessed by the Knaus Scale [12], baseline severity indexes, and simplified acute physiology score (SAPS) II [13] and Sequential Organ Failure Assessment (SOFA) [14] scores. A daily record was made throughout the ICU stay of clinical and biological parameters, administration of selective digestive decontamination (SDD), requirement for non-invasive and invasive oxygen therapy, other organ supports such as vasopressor use, renal replacement therapy (RRT), occurrence of ICU-acquired pneumonia and bacteremia, antibiotics administered, and the results of microbiological examinations. ICU and hospital length of stay, vital status at ICU and hospital discharge and at day 60 after ICU admission were recorded in patient files. Of note, SDD was very unfrequently used due to the level of antimicrobial resistance in the ICUs from the Outcome-REA database.

### Definitions

SARS-CoV-2 infection was identified by positive nasopharyngeal/throat swab or bronchoalveolar washings. Influenza infection was identified by positive throat swab, or respiratory or bronchoalveolar washings for influenza A or B.

IMV was defined as continuous ventilation via endotracheal tube or tracheotomy.

VAP was defined according to European guidelines by pulmonary parenchyma infection in patients exposed to IMV ≥ 48 h [15]. Quantitative cultures of low respiratory tract specimens were required to diagnose VAP. When bacteriological examinations yielded only coagulase-negative staphylococci or Enterococcus species, the diagnosis of VAP was only considered after careful checking by a senior investigator.

A further episode of VAP was considered to have occurred if a new infection was diagnosed 4 days after the previous episode of VAP.

Antimicrobial treatments were categorized for analysis in (1) amoxicillin/clavulanic acid, (2) ureido-carboxypenicillins/tazobactam, (3) third generation cephalosporins; (4) fourth generation cephalosporins, (5) carbapenems, (6) other ß-lactams consisting mainly of more recent ß-lactamase inhibitors such as ceftolozane/tazobactam or ceftazidime/avibactam, (7) aminoglycosides, (8) fluoroquinolones, and (9) macrolides.

Antibiotic treatment was considered adequate if one or more antibiotics initiated for VAP were active against the causative microorganism and administered at least within the first 24 h after VAP occurrence.

Early-onset VAP was defined as VAP occurring within the first 7 days of intubation, and late-onset as VAP occurring after the first 7 days of intubation.

High-risk pathogens were defined as multi-drug resistant bacteria and clustered into four classes: methicillin-resistant *Staphylococcus aureus* (MRSA), extended-spectrum beta-lactamase *Enterobacteriaceae* (ESBL), AmpC-producing *Enterobacteriaceae*, and *Pseudomonas aeruginosa* resistant to ticarcillin and/or imipenem and/or ceftazidime.

A patient was considered to be colonized if one of these four microorganisms were isolated from screening samples (perirectal, nose, or other) or from any other bacteriological samples issued of the usual care.

Co-infections included all the infections diagnosed on ICU admission.

### Endpoints

The primary outcome was to estimate the association between VAP and day-60 mortality in the whole cohort. The secondary objectives were to estimate the incidence of VAP in the two groups of patients, and to describe the etiologic microorganisms and outcome of VAP.

### Statistical analysis

Patient characteristics were expressed as *N* (%) for categorical variables and median (interquartile range (IQR)) for continuous variables. Comparisons were made with exact Fisher tests for categorical variables and Wilcoxon tests for continuous variables.

The type 1 incidence density was the ratio of the number of the first VAP episodes divided by the number of IMV days prior to the episode. The type 2 incidence density was the number of all VAP episodes divided by the number of IMV days during ICU stay. Incidence densities were compared by Poisson regression.

Multivariate analysis Cox survival models were used to determine the variables associated with day-60 mortality. The variables reaching a *p*-value < 0.2 in univariate analysis were tested in the multivariate model. The Bayesian information criterion (BIC) was estimated at each step of forward variable selection and was used as the metric to assess which factors were most influential in model prediction. Factors identified as important during forward variable selection were then further explored using a backward selection Cox survival model to determine their association with day-60 mortality. All terms with a p-value of < 0.05 remained in the backward selection survival model. VAP was handled as a delayed entry variable. VAP and COVID-19 were forced in the model. The interaction between VAP and COVID-19 was assessed in the whole cohort.

Similarly, using the same final model, we estimated the impact of early VAP versus non- early VAP and “late VAP without early VAP” versus “non-late VAP without early VAP” in the cohort of patients ventilated for at least 48 h.

In a sensitivity analysis to determine the patient characteristics to adjust for, we also used a Directed Acyclic Graph (DAG) to represent the direct causal effects of one patient characteristic on another [16, 17]. The patient characteristics we considered were based on the literature and availability in our data set: age, major comorbidities including obesity and immunodeficiency, time from symptoms to ICU admission, COVID, SOFA, antibiotic treatment, steroid, anti-inflammatory, antiviral treatment on admission, and SOFA and antimicrobial therapy on intubation. The identified factors that may confound the association between VAP and mortality were added to the Cox regression model as covariates.

A hazard ratio > 1 indicated an increased risk of day-60 mortality. The proportionality of hazard risks for covariates was assessed with martingale residuals. Continuous variables were dichotomized using the median or usual cut off value if necessary. To account for the multi-level of the data due to centres, random effect models with centres as a random variable were used.

For all tests, a two-sided α of 0.05 was considered to be significant. Missing baseline variables were handled by multiple imputation with only one dataset using proc MI with SAS software. All statistical analyses were performed with SAS software, Version 9.4 (SAS Institute, Cary, NC) and R software version 3.5.1 (http://www.R-project.org). The DAG was drawn and analyzed using the web application ‘DAGitty’ [http://dagitty.net/].

## Results

### Population

**Fig. 1:**
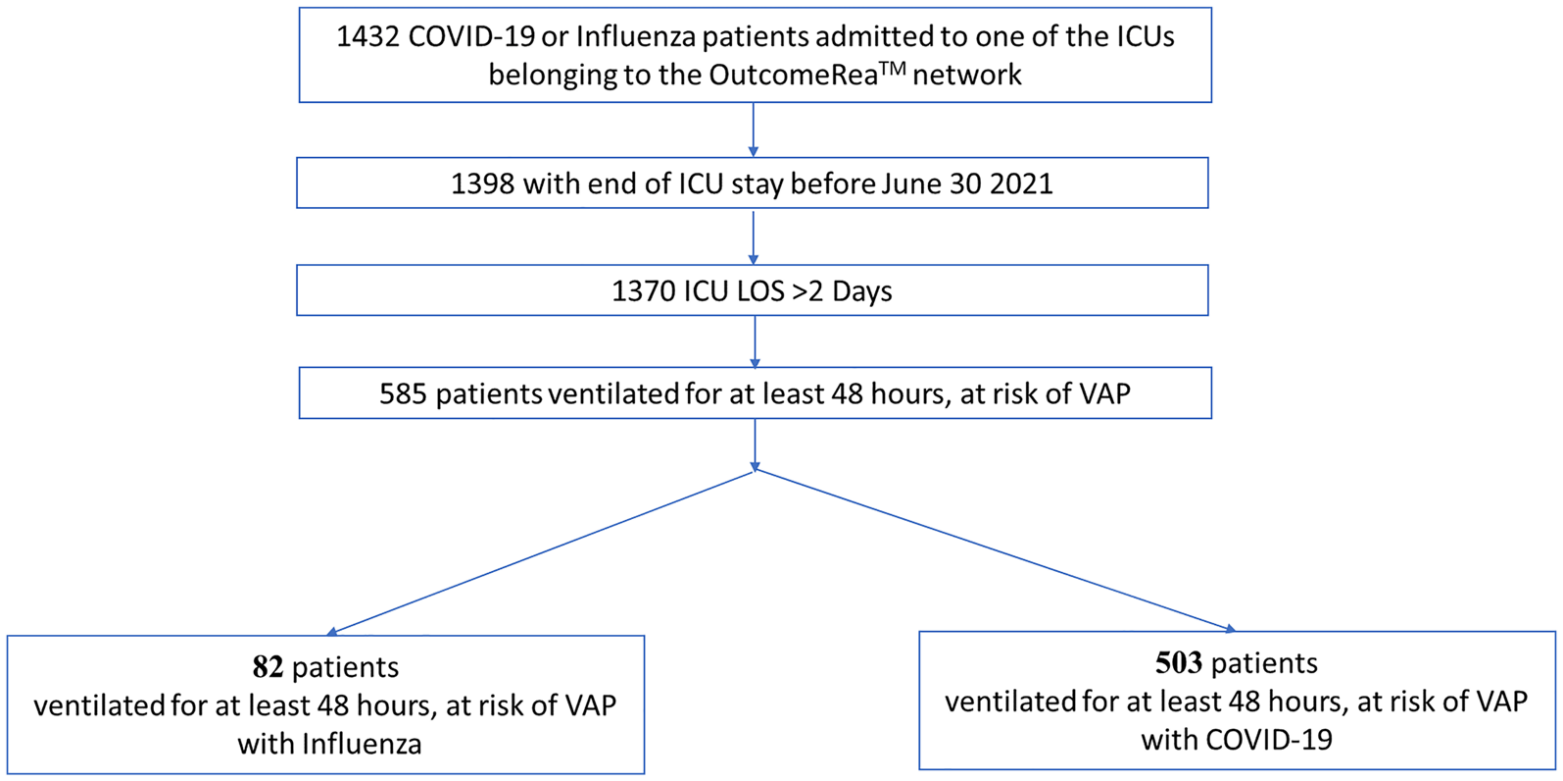
Flowchart. *ICU* intensive care unit, *LOS* length of stay, *VAP* ventilator-associated pneumonia

The study involved 585 mechanically ventilated patients for at least 48 h, 503 with COVID-19 and 82 with influenza (Fig. 1). The characteristics of the population are given in Tables 1, 2.

**Table 1:** Comparison between patients with influenza or COVID-19 pneumonia

Baseline characteristics (median [IQR] or N (%))	Influenza (*n* = 82)	COVID-19 (*n* = 503)	*p*-value
Period of admission			
Before 1 January, 2020	82 (100)	0 (0)	< 0.01
From 1January, 2020, to 31 July, 2020	0 (0)	278 (55.3)	
From 1 August, 2020 to 31 December, 2020	0 (0)	101 (20.1)	
From 1 January, 2021	0 (0)	124 (24.7)	
Age (years)	59 [51.4; 72]	64.3 [54.9; 72.6]	0.04
Gender (male)	46 (56.1)	369 (73.4)	< 0.01
Body-mass index ≥ 30 kg/m^2^	21 (25.6)	204 (40.6)	< 0.01
Chronic cardiac disease	8 (9.8)	138 (27.4)	< 0.01
Chronic respiratory disease	27 (32.9)	50 (9.9)	< 0.01
Chronic kidney disease	5 (6.1)	46 (9.1)	0.36
Immunosuppression^a^	26 (31.7)	52 (10.3)	< 0.01
Diabetes mellitus	14 (17.1)	89 (17.7)	0.89
Time between hospital and ICU admission	1 [1; 2]	2 [1; 4]	< 0.01
Severity and treatments on ICU admission			
SAPS II score	48 [36; 61]	38 [29; 50]	< 0.01
SOFA score	7 [5; 9]	7 [5; 9]	0.26
ARDS: PaO2/FiO2	127.6 [87; 198.7]	98.6 [68.8; 156]	< 0.01
Invasive mechanical ventilation	73 (89)	320 (63.7)	< 0.01
High-flow nasal cannula	7 (8.5)	172 (34.3)	< 0.01
Continuous positive airway pressure	11 (13.4)	54 (10.8)	0.48
ECMO	2 (2.4)	16 (3.2)	0.72
Prone position	8 (9.8)	115 (22.9)	< 0.01
RRT	7 (8.5)	28 (5.6)	0.30
Vasopressors	16 (19.5)	207 (41.2)	< 0.01
Antimicrobial treatment on admission	48 (58.5)	376 (74.8)	< 0.01
Amoxicillin and clavulanic acid	12 (14.6)	35 (7)	0.02
Ureido-carboxypenicillins	19 (23.2)	34 (6.8)	< 0.01
Third generation cephalosporins	28 (34.1)	270 (53.8)	< 0.01
Fourth generation cephalosporins	2 (2.4)	31 (6.2)	0.17
Penems	1 (1.2)	13 (2.6)	0.45
Macrolides	27 (32.9)	179 (35.7)	0.63
Aminoglycosides	10 (12.2)	41 (8.2)	0.23
Fluoroquinolones	6 (7.3)	32 (6.4)	0.75
Anti-MSSA*	3 (3.7)	5 (1)	0.05
Anti MRSA**	6 (7.3)	12 (2.4)	0.02
Lopinavir–Ritonavir	0 (0)	99 (19.7)	< 0.01
Hydroxychloroquine	0 (0)	34 (6.8)	0.02
Remdesivir	0 (0)	45 (8.9)	< 0.01
Ozeltamivir	30 (36.6)	22 (4.4)	< 0.01
Il1 R or Il 6 R antagonist	0 (0)	24 (4.8)	0.04
Steroids	25 (30.5)	262 (52.1)	< 0.01
Microbial colonization on admission	7 (8.5)	25 (5)	0.45
Bacterial pneumonia co-infection on admission	27 (32.9)	56 (11.1)	< 0.01

SAPS II simplified acute physiology score, SOFA sequential organ failure assessment, ARDS acute respiratory distress syndrome, ECMO extra corporeal membrane oxygenation, RRT renal replacement therapy, MSSA methicillin-susceptible *Staphylococcus aureus*, MRSA methicillin-resistant *Staphylococcus aureus*

^a^Organ transplants, AIDS, non-AIDS HIV, corticoids > 1 month or > 2 mg/kg/j, chemotherapy, aplasia, or other immunodepression.^*^cefazolin, or penicillin; ^**^linezolid, daptomycin, vancomycin

**Table 2:** Characteristics before the risk of VAP, during ICU stay and main outcomes

Characteristics (median [IQR] or *N*(%))	Influenza (*n* = 82)	COVID-19 (*n* = 503)	*p*-value
Timing from ICU admission to intubation	1 [1; 2]	2 [1; 3]	< 0.01
Treatments during ICU stay			
Selective digestive decontamination^a^	0 (0)	54 (10.7%)	< 0.01
Prone position	18 (22)	262 (52.1)	< 0.01
RRT	24 (29.3)	161 (32)	0.62
ECMO	5 (6.1)	54 (10.7)	0.20
Vasopressors	19 (23.2)	341 (67.8)	< 0.01
Nosocomial infections during ICU stay			
Bacteremia	16 (19.5)	108 (21.5)	0.69
Fungemia	2 (2.4)	48 (9.5)	0.03
VAP	23 (28)	209 (41.6)	0.02
Early VAP (during the first 7 days)	6 (7.3)	85 (16.9)	0.03
Late VAP (after day 7)	19 (23.2)	161 (32)	0.11
Main outcome measures			
Duration of invasive mechanical ventilation	12 [6; 22]	13 [7; 23]	0.41
Duration of RRT	9 [4.5; 16.5]	10 [3; 18]	0.90
Duration of ECMO	3 [1; 4]	12.5 [6; 19]	0.05
Ventilatory-free days at day 28	11 [0; 21]	0 [0; 15]	< 0.01
RRT-free days at day 28	28 [15; 28]	25 [0; 28]	< 0.01
ECMO-free days at day 28	28 [28; 28]	28 [0; 28]	< 0.01
ICU LOS	14.5 [9; 28]	16 [10; 28]	0.37
Hospital LOS	30.5 [13; 48]	22 [14; 40]	0.11
Mortality at day 60	19 (23.2)	233 (46.3)	< 0.01

VAP ventilator-associated pneumonia, ICU intensive care unit, RRT renal replacement therapy, ECMO extra corporeal membrane oxygenation, LOS length of stay

^a^Without intravenous antimicrobial therapy

### Baseline characteristics

Patients with SARS-CoV-2 pneumonia were older than patients with influenza, 64.3 [54.9; 72.6] vs 59 [51.4; 72], (*p *< 0.01); more often obese, 40.6% vs 25.6%, (*p* < 0.01); more prone to cardiac diseases 27.4% vs 9.8%, *p* < 0.01); less frequently immunocompromised, 10.3% vs 31.7%, (*p* < 0.01); and had lower rates of chronic respiratory failure, 9.9% vs 32.9% (*p* < 0.01).

### Characteristics on admission

On ICU admission, SARS-CoV-2 pneumonia patients had a lower SAPS II score than influenza pneumonia patients, 38 [29; 50] vs 48 [36; 61] (*p* < 0.01); were more severely hypoxemic, P/F ratio = 98.6mmHg [68.8; 156] vs 127.6 [87; 198.7], (*p* < 0.01); and less frequently placed on invasive mechanical ventilation, 63.7% vs 89%, (*P* < 0.01), respectively.

### Microbiological results and antimicrobial treatment at the time of admission

On ICU admission, patients with SARS-CoV-2 pneumonia had a lower rate of pulmonary bacterial co-infections than influenza pneumonia patients, 11.1% vs 32.9%, (*p* < 0.01); more frequent administration of antibiotics, 74.8% vs 58.5%, (*p* < 0.01) and of corticosteroids, 52.1% vs 30.5%, (*p* < 0.01), respectively. Only patients with COVID-19 received IL-6 or Il 1 receptor antagonist treatments, 4.8% vs 0% (*p* < 0.01), respectively.

### During ICU stay

During ICU stay, patients with SARS-CoV-2 pneumonia compared with patients with Influenza were more often under vasopressors: 67.8% vs 23.2%, (*p* < 0.01), respectively; but no difference in RRT or ECMO requirement was observed between the two groups of patients. Only patients with COVID-19 received SDD: 10.7% vs 0% (*p* < 0.01), respectively.

There was a trend to a greater ICU length of stay, and a shorter hospital length of stay between SARS-CoV-2 pneumonia patients and Influenza pneumonia patients: 16 days [10; 28] vs 14.5 [9; 28], (*p* = 0.1); and 22 [14; 40] vs 30.5 [13; 48] (*p* = 0.11); respectively. Day-60 mortality rate was twice higher in COVID-19 patients than in flu patients: 46.3% vs 23.2% (*p* < 0.01), respectively.

### Ventilator associated pneumonia

**Fig. 2:**
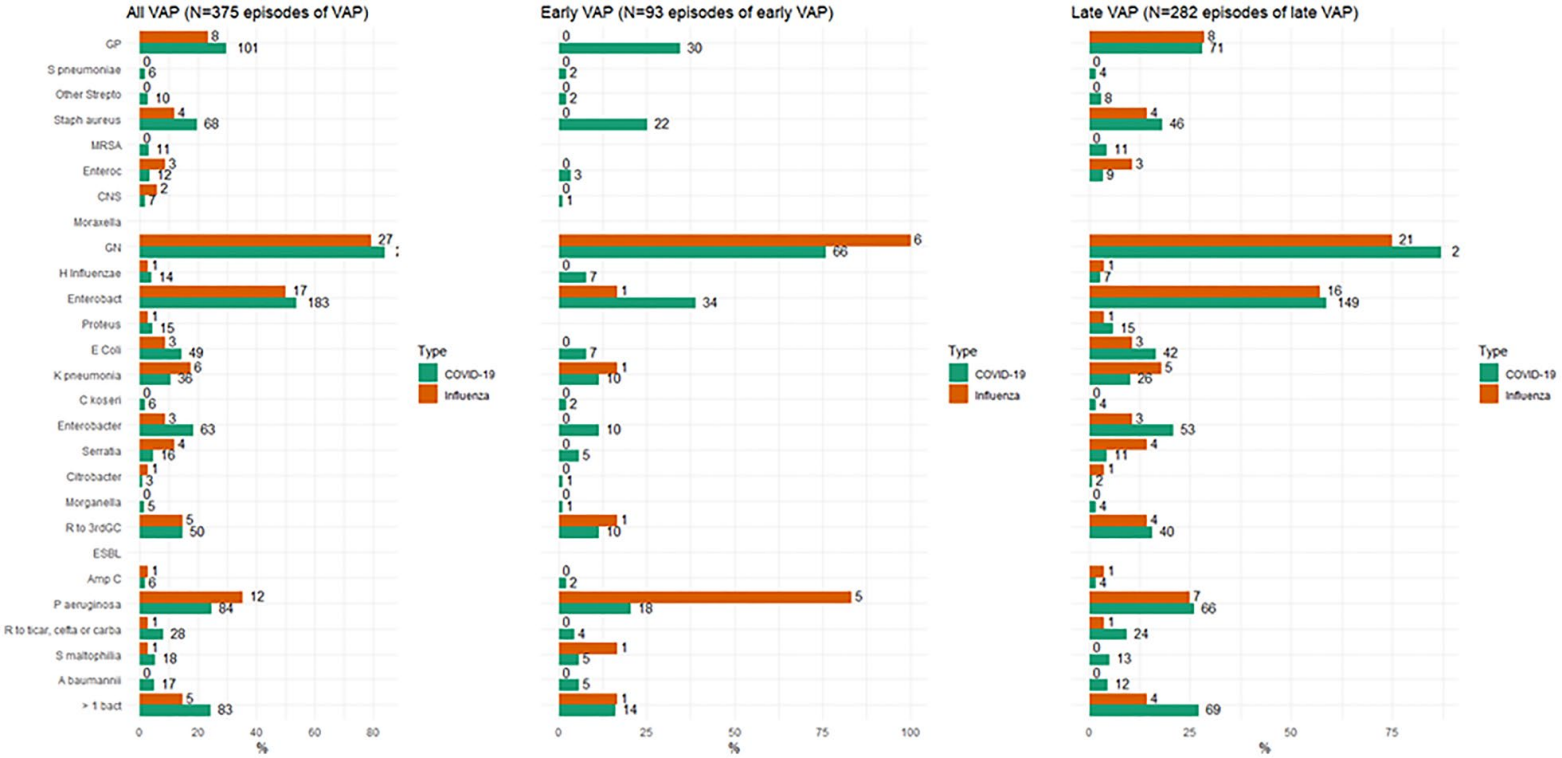
Microbiological characteristics of VAP, early and late VAP for patients with COVID or influenza. GP = Gram-positive; MRSA: Methicillin resistant *Staphylococcus* *aureus*, *Enterococ*: Enterococcus, *CNS* coagulase negative *Staphylococcus*, *GN* gram negative, *Enterobact* Enterobacteriaceae, *R to 3rdGC* Resistant to third generation cephalosporin, *ESBL* Extended spectrum beta lactamase, *AmpC *Derepressed cephalosporinase, R to ticar, cefta or carba: Resistant to ticarcillin, ceftazidime or carbapenems; > 1 bact: More than one bacteria

In COVID-19 and influenza patients, respectively, 209 (41.6%) and 23 (28%) (*p* < 0.01) developed 341 and 34 episodes of VAP. Type 1 incidence was 99.2 and 56.4 per 1000 IMV-days (*p* < 0.01), respectively, and type 2 incidence 32.8 and 17.8 VAP per 1000 IMV-days (*p* < 0.01). The total number of isolated etiologic microorganisms was 375. In COVID-19 and influenza patients, respectively, the microorganisms were Gram positive cocci (GPC) in 29.6% and 23.5% of cases, with methicillin-susceptible *Staphylococcus aureus* being the predominant microorganism in 19.9% and 11.8% of patients. Similarly, the microorganisms were, respectively, Gram-negative bacilli (GNB) in 84.2% and 79.4%, with *Enterobacteriaceae* being the predominant microorganism in 63% and 62%, followed by *Pseudomonas aeruginosa* in 24.6% and 35.3%. Only marginal differences in the etiologic microorganisms of VAP were observed between COVID-19 and flu. The distribution of microorganisms responsible for VAP is shown in Fig. 2 and Additional file 1: Table S1.

### Comparison of patients with and without VAP among patients with COVID-19 and those with influenza

(Additional file 1: Table S2) Among COVID-19 patients, VAPs patients had fewer chronic cardiovascular and immunosuppression comorbidities compared with those without VAP. On admission, VAP patients were more severely hypoxic, were more often administered corticosteroids and less frequently antibiotics. During ICU stay, VAP patients needed more frequently prone positioning and RRT. They had a longer ICU and hospital length of stay but a non-different day-60 mortality rate (Additional file 1: Table S2).

Among influenza patients, no difference was observed in baseline characteristics between VAP and non-VAP patients. VAP patients had a longer duration of IMV, and a trend to a higher requirement of RRT and extracorporeal membrane oxygenation (ECMO) compared to non-VAP patients. VAP patients had a longer duration of IMV, and ICU and hospital length of stay, but a non-different day-60 mortality rate (Additional file 1: Table S2).

### Comparison between COVID-19 and influenza patients among patients with VAP and those without

(Table 3 and Additional file 1: S2) In patients without VAP, the differences observed between COVID-19 and influenza patients were similar to those observed between the two groups in the whole cohort.

**Table 3:** Main outcome measures

	All			Influenza			COVID-19		
	No VAP	VAP	*p*-value	No VAP	VAP	*p*-value	No VAP	VAP	*p*-value
Number of patients	353	232		59	23		294	209	
Duration of IMV	9 [5; 14]	20.5 [14; 33]	< 0.01	8 [4; 17]	22 [14; 40]	< 0.01	9 [5; 14]	20 [14; 33]	< 0.01
Duration of RRT	7 [3; 14]	15 [7; 25]	< 0.01	9 [4; 14]	9 [9; 84]	0.35	6 [3; 13]	15 [6; 25]	< 0.01
Duration of ECMO	6 [3; 13]	15 [8; 25]	< 0.01	3.5 [2; 11.5]	1 [1; 1]	0.51	7 [3; 13]	15 [8; 25]	0.01
Ventilatory-free days at day 28	0 [0; 0]	0 [0; 0]	1.00	0 [0; 0]	0 [0; 0]	1.00	0 [0; 0]	0 [0; 0]	1.00
RRT-free days at day 28	0 [0; 28]	28 [0; 28]	0.50	28 [0; 28]	28 [28; 28]	0.05	0 [0; 28]	0 [0; 28]	0.82
ECMO-free days at day 28	28 [0; 28]	28 [0; 28]	0.49	28 [28; 28]	28 [28; 28]	0.53	28 [0; 28]	28 [0; 28]	0.32
ICU LOS	13 [8; 19]	25 [16; 38]	< 0.01	13 [7; 20]	27 [19; 45]	< 0.01	13 [8; 19]	25 [16; 38]	< 0.01
Hospital LOS	18 [11; 31]	34 [19; 51.5]	< 0.01	21 [13; 41]	46 [35; 57]	< 0.01	17 [11; 30]	31 [19; 50]	< 0.01
Day-60 mortality	149 (42.2)	103 (44.4)	0.60	15 (25.4)	4 (17.4)	0.44	134 (45.6)	99 (47.4)	0.69

*VAP* ventilator-associated pneumonia, *IMV* Invasive mechanical ventilation, *RRT* renal replacement Therapy, *ECMO* extra-corporeal membrane oxygenation, *ICU* intensive care unit, *LOS* length of stay

In the VAP group, COVID-19 patients were more frequently males than flu patients, had a higher body mass index, less frequently chronic liver or chronic respiratory disease and immunosuppression, were admitted to ICU after a longer hospital stay, had more frequently a co-infection with bacterial pneumonia, were more frequently treated by antibiotics, corticosteroids, high flow nasal cannula, prone positioning, nitric oxide, ECMO and vasopressors. They had a longer duration of IMV, and a higher day-60 mortality rate.

### Risk of day-60 mortality due to VAP

**Fig. 3:**
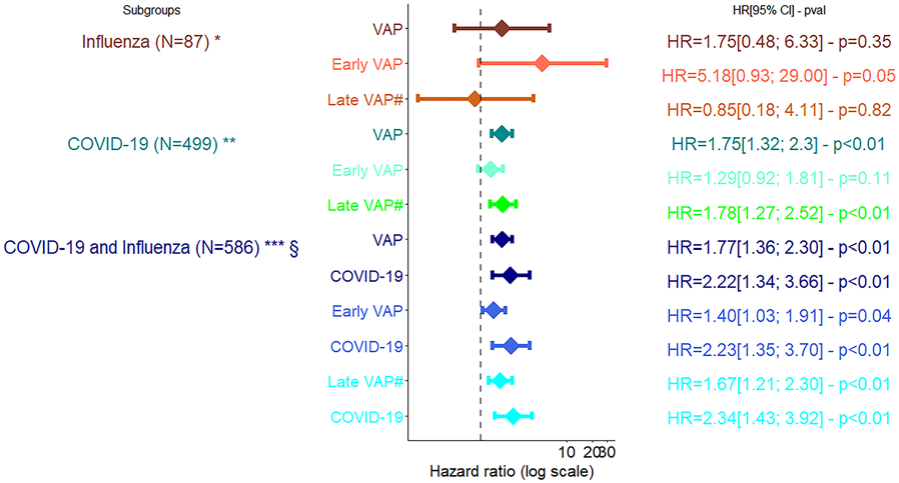
Association between VAP and day-60 mortality among patients with COVID and/or influenza, multivariate Cox model. *Adjustment for age, immunosuppression, steroids on admission, renal SOFA before VAP. **Adjustment for age, comorbidities, immunosuppression, time from admission to intubation > 5 days, time from symptoms to ICU admission > 10 days, Broad spectrum antimicrobial therapy, Renal SOFA > 2, ECMO, parenteral feeding. ***Adjustment for age, comorbidities, immunosuppression, time from admission to intubation > 5 days, time from symptoms to ICU admission > 10 days, Cardio SOFA > 2, Renal SOFA > 2, blocking agent, parenteral feeding, §interactions were tested and there was no interaction between VAP and COVID-19; early VAP and COVID-19 and late VAP and COVID-19. #Late VAP without early VAP *VAP* ventilator associated pneumonia

**Fig. 4:**
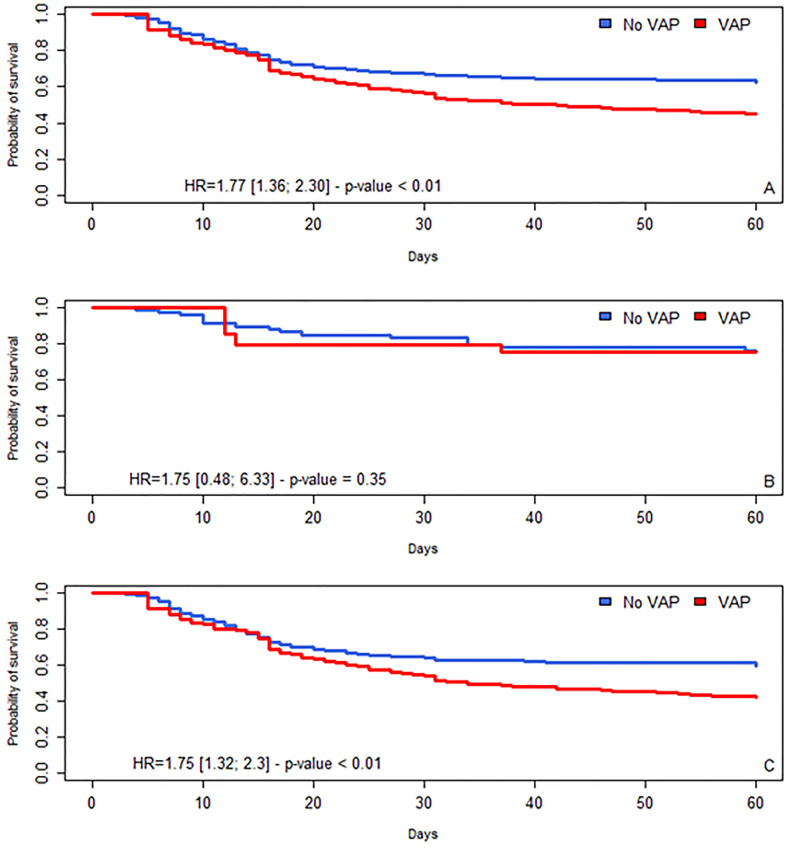
Cumulative likelihood of survival over time in patients with and without VAP. VAP: ventilator-associated pneumonia, HR = hazard ratio (with 95% confidence interval). Day 0 indicates the date of intubation. **A**: all patients; **B**: patients with influenza, **C**: patients with COVID-19

(Table 3, Additional file 1: Table S3, S4 and Figs. 3, 4) In the whole cohort, COVID-19 patients were independently associated with an increased risk of death compared with influenza patients (aHR = 1.77 [1.36; 2.30], *p* < 0.01). In the whole cohort, VAP was independently associated with an increased risk of death (aHR = 2.22 [1.34; 3.66], *p* < 0.01). Similar results were observed for early-onset and late-onset VAP. There was no interaction between the occurrence of VAP and type of virus on admission. Even with adequate antimicrobial therapy, VAP was still associated with an increased risk of death (aHR = [1.15; 2.17], *p* < 0.01).

Among COVID-19 patients ventilated for at least 48 h, VAP was associated with an increased risk of day-60 mortality (aHR = 1.75 [CI 95%, 1.32; 2.33], *p* < 0.01), and only late VAP increased the risk (aHR = 2.05 [CI 95%, 1.48; 2.85], *p* < 0.01). Even with adequate antimicrobial therapy, VAP was still associated with an increased risk of death (aHR = 1.63[1.18; 2.25], *p* < 0.01).

Among influenza patients ventilated for at least 48 h, only early VAP was marginally associated with a higher risk of day-60 mortality (aHR = 5.18 [CI 95%, 0.93; 29], p = 0.055). Similar results were found selecting covariates using DAG (Additional file 1: Table S5, Fig S1).

## Discussion

We compared patients ventilated either for COVID-19 or for influenza, at risk of VAP, i.e., patients ventilated for at least 48 h, particularly in terms of VAP incidence, microbiology, and outcome.

The main findings were: (1) a higher incidence of VAP in the group of patients with COVID-19 than in the group with influenza, (2) in both COVID-19 and influenza patient groups, the etiologic microorganisms were mostly GBN, (3) in the COVID-19 group, VAP and particularly late-onset VAP were associated with an increased risk of death, while in the influenza group only early-onset VAP tended to be associated with this risk, and (4) in the whole cohort, VAP, and COVID-19, as compared with influenza, were associated with an increased risk of death with similar results being observed for early- and late-onset VAP.

In patients receiving MV for other causes than COVID-19, the incidence of VAP ranged from 12 to 28 VAP per 1000 ventilation days, which is consistent with our results in the influenza group [18, 19]. Additionally, in our work, the incidence of VAP in the COVID-19 group is in line with previously published data for this population. Like most authors, we found a higher rate of VAP among COVID-19 patients than in patients receiving MV for causes other than COVID-19 [20–23].

The higher rates of VAP observed in COVID-19 patients could have been due to over-exposure to immunomodulatory treatments including corticosteroids and anti-interleukin receptors. Other possible explanations include: (1) an uncontrolled immune response triggered by SARS-CoV2 itself leading to a more severe acute respiratory failure with lower PaO2/FiO2 and a more frequent need for higher Peep and prone positioning resulting in prolonged use of sedation and longer duration of mechanical ventilation; (2) less rigorous practice of standard prevention strategies during COVID-19; (3) an increased use of endotracheal aspirates for VAP diagnosis, a technique that is more sensitive than broncho-alveolar lavage for VAP diagnosis [23]; (4) difficulties in distinguishing trachea bronchitis from pneumonia; (5) the higher rate and severity of comorbidities often observed in COVID-19 patients requiring MV [18, 24–26]; (6) higher risk for pulmonary infarction; and (7) overcrowding often combined with understaffing of both staff and physicians during the COVID pandemic [24].

We also observed an association between VAP and increased risk of death, but only in COVID-19 patients. Whether VAP results in higher ICU or hospital mortality is debated. In several of the few studies dealing with the topic over the past 15 years, VAP was not associated with increased ICU mortality, or had only a marginal effect on the outcome [27–30]. In most studies, the main morbidity due to VAP was a delay in extubation and an increased ICU and hospital length of stay [31, 32]. However, in more recent and thorough studies like those of Melsen et al. [33] and Boyd et al. [5], VAP in the ICU setting was associated with an increased death rate. In our study, we observed an increased risk of death associated with VAP but only in COVID-19 patients. Such a result was previously obtained, for instance, by Mussuza et al. who reported a higher odds of death in COVID-19 patients with co- or super-infections (odds ratio = 3.31, 95% CI 1.82–5.99) [20]. The main causes are unclear and need further investigation. The impaired pulmonary microbiome and local immune defenses in acute respiratory distress syndrome (ARDS) with a cytokine storm could be a key element in understanding the severity of the syndrome and the poorer outcome of secondary superinfection in mechanically ventilated patients. ARDS could be more severe in COVID-19 patients [34], and VAP in itself could worsen the pre-morbid conditions that lead to an increased risk of death.

It emerged from our study that VAP increased the risk of death. It is therefore extremely important to prevent the risk of VAP and improve patient treatment. Hence, all the practices recommended or proposed to prevent VAP should be carefully applied in COVID-19 patients. They include reducing exposure duration to mechanical ventilation, improving hand hygiene compliance, inclining the head of the bed, oral care, weaning sedation, draining subglottic secretion, early weaning from ventilation, and early promotion of patient mobilization [35–38]. In addition, favouring non-invasive ventilation and avoiding early invasive intubation have been suggested by recent studies [39].

To improve the treatment of VAP in COVID-19 patients, early diagnosis is of major importance. Unfortunately, standard clinical diagnostic criteria for VAP are invalid in the critical COVID-19 population [40, 41]. Against this background, shortening the time from sampling to pathogen identification, and the use of multiplex PCR pneumonia panels have been proposed in COVID-19 patients to improve detection of VAP [24, 42]. Research is now in progress on the use of procalcitonin (PCT) to distinguish between viruses and bacteria as etiologic agents of VAP [43, 44]. Antimicrobial stewardship with the appropriate type, timing and duration of antibiotics for VAP is also crucial, and avoiding inappropriate empiric antibiotic use is now recognized as a priority in the management of COVID-19 patients [45].

Our results showed that when patients with acute hypoxemic respiratory failure due to either COVID-19 or influenza were considered together, COVID-19 by itself was associated with a higher risk of mortality, and that the same result was observed when the cohort was restricted to patients without VAP. Similar results were reported by Cobb et al. [46]. The higher risk of mortality observed in COVID-19 than in influenza could be due first to the higher rate of actual ARDS seen in COVID-19 than in influenza, which results in both longer MV duration and ICU length of stay [34], and second to a difference in the host immune response between COVID-19 and influenza patients with, in the former, a greater cytokine storm and more frequent severe organ damage such as acute kidney injury and vasopressors use [47, 48]. It is also likely that in influenza patients, the extensive administration of oseltamivir in the initial phase of the disease attenuates the intensity of the lesions by accelerating viral clearance. Finally, some of the patients included in the COVID-19 group were managed at the beginning of the first wave of the pandemic and did not receive corticosteroids or reinforced preventive and/or curative anticoagulation, which have demonstrated their effectiveness in improving prognosis in this patient category [49].

The distribution of etiologic microorganisms of VAP in COVID-19 patients differs between reports in the literature. In our study, *Enterobacteriaceae* were the most frequent causative agents, in agreement with several recent published articles [22, 50–57]. However, in another study etiologic agents were mainly GPC [9]. We also observed that bacterial co-infections during VAP were frequent in COVID-19 patients.

The main hypothesis, other than a change in pulmonary microbiota composition due to corticosteroids or viruses, is the high prescription rate of anti-GPC regimens at the time of admission to the ICU, which could have modified the results of respiratory microbiological sampling. Early-onset VAP is classically due to pathogens of the normal oropharyngeal flora buts late-onset VAP to GBN with a high prevalence of multi-drug resistant pathogens [18, 25]. In studies on VAP and COVID-19, the prevalence of GBN, comprising mainly *Enterobacteriaceae* in 5% to 70% of cases, ranged between 40 and 83.6% [34], and non-fermenting GNB in 17% to 40% of cases, chiefly *Pseudomonas aeruginosa* but also *Acinetobacte*r spp [20]. The prevalence of GPC, in most cases *S. aureus,* ranged between 3 and 36%. A quite high proportion of multi-drug resistant isolates were reported ranging from 23 to 67%, with MRSA in 1.5% to 24% of patients [21].

The main strengths of this study are the large sample size of the population recruited in a network of 11 ICUs, and prospective data collection in a high-quality database that allowed accurate assessment of patients with influenza and COVID-19.

The limitations are the imbalance in the number of patients between the COVID-19 and influenza groups; the difference in recruitment periods between the two groups, both of which, however, were treated according to the standard of care of high-income countries; the change in non-invasive oxygen therapy strategies over time such as the recent extensive use of high flux nasal oxygen therapy in hypoxemic pneumonia; the absence of a standardized protocol for respiratory microbiological sampling across centres (but quantitative microbiological cultures of bronchoalveolar lavages and of tracheal aspirates were performed in all centres throughout the study period to diagnose VA); and recruitment of most COVID-19 patients during the first wave, i.e., before the systematic use of corticosteroids, which was shown to improve the prognosis of COVID-19 in hypoxemic patients. Some biomarkers to predict the occurrence of VAP such as serum levels of C-reactive proteins and of procalcitonin were lacking in our cohort mostly for influenza patients, and these covariates could not therefore be considered in the various models. All influenza patients were admitted before COVID-19 patients, and so we were unable to adjust for the study period by dividing it up. However, the definitions and treatments of VAP were similar throughout the study period, which minimized the bias due to the absence of adjustment for this covariate. Thus, our model, based on a multivariable Cox model, was only able to estimate association and not causality. However, after selection of our covariates by DAG, we obtained similar results. Taken together, these limitations preclude any direct comparison of the impact of VAP between the two groups.

## Conclusion

The incidence of VAP was higher in patients at risk of VAP who had been ventilated for at least 48 h for COVID-19 but not for influenza. VAP was mostly associated with an increased risk of death in COVID-19 patients. A standardized multimodal VAP prevention approach as classically recommended should be applied to all patients at risk of VAP. New strategies should be investigated to complement these preventive measures to decrease the higher rate of VAP in the COVID-19 population.

### Supplementary Information

**Additional file 1: Table S1.** Microbiological characteristics of early and late VAP in patients with COVID and influenza. **Table S2.** Comparison between Influenza and COVID patients depending on the occurrence of the first episode of VAP. **Table S3.** Risk factors of day-60 mortality in the whole cohort—Univariate analyses—Survival Cox models. **Table S4.** Association between VAP and day-60 mortality—multivariate survival Cox models. **Table S5.** Factors associated with day-60 death, multivariate cox model, section of the covariates with DAG. **Figure S1.** Directed Acyclic Graph—**A** Unadjusted and **B** adjusted

## Data Availability

Data can be provided upon request to the corresponding author.

## Abbreviations

aHR: Adjusted hazard ratio
ARDS: Acute respiratory distress syndrome
BIC: Bayesian information criterion
CCTIRS: Comité Consultatif sur le Traitement de l’Information en matière de Recherche dans le domaine de la Santé
CI: Confidence interval
CNIL: Commission Nationale de l'Informatique et des Libertés
DAG: Directed acyclic graph
ECMO: Extra corporeal membrane oxygenation
ESBL: Extended-spectrum beta-lactamase enterobacteriaceae
GNB: Gram negative bacilli
GPC: Gram positive cocci
HR: Hazard ratio
ICU: Intensive care unit
IMV: Invasive mechanical ventilation
IQR: Interquartile range
IRB: Institutional review board
MRSA: Methicillin-resistant *staphylococcus aureus*
SAPS: Simplified acute physiology score
SOFA: Sequential organ failure assessment
VAP: Ventilated acquired pneumonia
