# Supplementary material for: Mortality, incidence, and microbiological documentation of ventilated acquired pneumonia (VAP) in critically ill patients with COVID-19 or influenza
Source: Ann Intensive Care. 2023 Oct 30;13:108. doi: 10.1186/s13613-023-01207-9 (PMC10616026; doi:10.1186/s13613-023-01207-9)
Supplement: Supplementary file 1 — Additional file 1: Table S1. Microbiological characteristics of early and late VAP in patients with COVID and influenza. Table S2. Comparison between Influenza and COVID patients depending on the occurrence of the first episode of VAP. Table S3. Risk factors of day-60 mortality in the whole cohort—Univariate analyses—Survival Cox models. Table S4. Association between VAP and day-60 mortality—multivariate survival Cox models. Table S5. Factors associated with day-60 death, multivariate cox model, section of the covariates with DAG. Figure S1. Directed Acyclic Graph—A Unadjusted and B adjusted [file 13613_2023_1207_MOESM1_ESM.docx]

**Additional file**

**Mortality, incidence, and microbiological documentation of ventilated acquired pneumonia in COVID19 and influenza patients at risk of VAP in ICU settings.**

Table S 1: Microbiological characteristics of early and late VAP in patients with COVID and influenza

|  | All VAP episodes | | | Early VAP episodes | | | Late VAP episodes | | |
| --- | --- | --- | --- | --- | --- | --- | --- | --- | --- |
| Variables | **Influenza** | **COVID-19** | **p-value** | **Influenza** | **COVID-19** | **p-value** | **Influenza** | **COVID-19** | **p-value** |
| Number of VAP episodes | 34 | 341 | . | 6 | 87 | . | 28 | 254 | . |
| Gram positive cocci | 8 (23.5) | 101 (29.6) | 0.46 | 0 (0) | 30 (34.5) | 0.08 | 8 (28.6) | 71 (28) | 0.94 |
| Streptococcus pneumoniae | 0 (0) | 6 (1.8) | 0.44 | 0 (0) | 2 (2.3) | 0.71 | 0 (0) | 4 (1.6) | 0.50 |
| Other Streptococci | 0 (0) | 10 (2.9) | 0.31 | 0 (0) | 2 (2.3) | 0.71 | 0 (0) | 8 (3.1) | 0.34 |
| Staphyloccus aureus | 4 (11.8) | 68 (19.9) | 0.25 | 0 (0) | 22 (25.3) | 0.16 | 4 (14.3) | 46 (18.1) | 0.62 |
| Methicillin resistant | 0 (0) | 11 (3.2) | 0.29 |  |  | . | 0 (0) | 11 (4.3) | 0.26 |
| Enterococcus | 3 (8.8) | 12 (3.5) | 0.13 | 0 (0) | 3 (3.4) | 0.64 | 3 (10.7) | 9 (3.5) | 0.07 |
| Staphyloccus coagulase negative | 2 (5.9) | 7 (2.1) | 0.16 | 0 (0) | 1 (1.1) | 0.79 | 2 (7.1) | 6 (2.4) | 0.15 |
| Moraxella |  |  | . |  |  | . |  |  | . |
| Gram negative bacteria | 27 (79.4) | 287 (84.2) | 0.47 | 6 (100) | 66 (75.9) | 0.17 | 21 (75) | 221 (87) | 0.08 |
| Haemophilus influenzae | 1 (2.9) | 14 (4.1) | 0.74 | 0 (0) | 7 (8) | 0.47 | 1 (3.6) | 7 (2.8) | 0.81 |
| Enterobacteriaceae | 17 (50) | 183 (53.7) | 0.68 | 1 (16.7) | 34 (39.1) | 0.27 | 16 (57.1) | 149 (58.7) | 0.88 |
| Proteus | 1 (2.9) | 15 (4.4) | 0.69 |  |  | . | 1 (3.6) | 15 (5.9) | 0.61 |
| Escherichia coli | 3 (8.8) | 49 (14.4) | 0.37 | 0 (0) | 7 (8) | 0.47 | 3 (10.7) | 42 (16.5) | 0.42 |
| Klebsiella pneumonia | 6 (17.6) | 36 (10.6) | 0.21 | 1 (16.7) | 10 (11.5) | 0.70 | 5 (17.9) | 26 (10.2) | 0.22 |
| Citrobacter koseri | 0 (0) | 6 (1.8) | 0.44 | 0 (0) | 2 (2.3) | 0.71 | 0 (0) | 4 (1.6) | 0.50 |
| Enterobacter | 3 (8.8) | 63 (18.5) | 0.16 | 0 (0) | 10 (11.5) | 0.38 | 3 (10.7) | 53 (20.9) | 0.20 |
| Serratia | 4 (11.8) | 16 (4.7) | 0.08 | 0 (0) | 5 (5.7) | 0.55 | 4 (14.3) | 11 (4.3) | 0.03 |
| Citrobacter | 1 (2.9) | 3 (0.9) | 0.26 | 0 (0) | 1 (1.1) | 0.79 | 1 (3.6) | 2 (0.8) | 0.17 |
| Morganella | 0 (0) | 5 (1.5) | 0.48 | 0 (0) | 1 (1.1) | 0.79 | 0 (0) | 4 (1.6) | 0.50 |
| Resistant to third generation cephalosporin | 5 (14.7) | 50 (14.7) | 0.99 | 1 (16.7) | 10 (11.5) | 0.70 | 4 (14.3) | 40 (15.7) | 0.84 |
| Extended spectrum beta lactamase | 3 (8.8) | 7 (2.1) | 0.02 | 1 (16.7) | 0 (0) | <.01 | 2 (7.1) | 7 (2.8) | 0.21 |
| Derepressed cephalosporinase | 1 (2.9) | 6 (1.8) | 0.63 | 0 (0) | 2 (2.3) | 0.71 | 1 (3.6) | 4 (1.6) | 0.45 |
| Pseudomonas aeruginosa | 12 (35.3) | 84 (24.6) | 0.17 | 5 (83.3) | 18 (20.7) | <.01 | 7 (25) | 66 (26) | 0.91 |
| Resistant to ticarcillin, ceftazidime or carbapenems | 1 (2.9) | 28 (8.2) | 0.27 | 0 (0) | 4 (4.6) | 0.59 | 1 (3.6) | 24 (9.4) | 0.30 |
| Stenotrophomonas maltophilia | 1 (2.9) | 18 (5.3) | 0.55 | 1 (16.7) | 5 (5.7) | 0.29 | 0 (0) | 13 (5.1) | 0.22 |
| Acinetobacter baumannii | 0 (0) | 17 (5) | 0.18 | 0 (0) | 5 (5.7) | 0.55 | 0 (0) | 12 (4.7) | 0.24 |
| More than one bacterium | 5 (14.7) | 83 (24.3) | 0.21 | 1 (16.7) | 14 (16.1) | 0.97 | 4 (14.3) | 69 (27.2) | 0.14 |

Table S 2: Comparison between Influenza and COVID patients depending on the occurrence of the first episode of VAP.

| Baseline characteristics | 1. Inf/VAP- | 2. Inf/VAP+ | 3. COV/VAP- | 4. COV/VAP+ | p-value |
| --- | --- | --- | --- | --- | --- |
| Number of patients | 59 | 23 | 294 | 209 | All |
| Period of admission |  |  |  |  |  |
| Before 1 January, 2020 | 59 (100) | 23 (100) |  |  | <0.01 |
| From 1January, 2020, to 31July, 2020 |  |  | 167 (56.8) | 111 (53.11) | . |
| From 1 August, 2020 to 31December, 2020 |  |  | 54 (18.37) | 47 (22.49) | . |
| From 1 January, 2021 |  |  | 73 (24.83) | 51 (24.4) |  |
| Age (years) | 57.71 [46.36 ; 72.22] | 59.24 [54.83 ; 72] | 65.77 [55.89 ; 73.05] | 63.4 [53.73 ; 71.66] | 0.04^c^ |
| Gender (male) | 34 (57.63) | 12 (52.17) | 208 (70.75) | 161 (77.03) | <0.01^cd^ |
| Body-mass index ≥ 30 kg/m² | 15 (25.42) | 6 (26.09) | 110 (37.41) | 94 (44.98) | 0.02 |
| Comorbidities |  |  |  |  |  |
| Chronic Liver Failure | 2 (3.39) | 3 (13.04) | 7 (2.38) | 2 (0.96) | <0.01^d^ |
| Chronic Cardiovascular Disease | 6 (10.17) | 2 (8.7) | 91 (30.95) | 47 (22.49) | <0.01^bc^ |
| Chronic Respiratory Failure | 19 (32.2) | 8 (34.78) | 30 (10.2) | 20 (9.57) | <0.01^cd^ |
| Chronic Kidney Disease | 5 (8.47) | 0 | 32 (10.88) | 14 (6.7) | 0.17 |
| Immunosuppression§ | 22 (37.29) | 4 (17.39) | 40 (13.61) | 12 (5.74) | <0.01^bcd^ |
| Diabetes | 9 (15.25) | 5 (21.74) | 52 (17.69) | 37 (17.7) | 0.92 |
| Time between hospital and ICU admission | 1 [1 ; 2] | 1 [1 ; 3] | 2 [1 ; 4] | 2 [1 ; 4] | <0.01^cd^ |
| Characteristics on admission |  |  |  |  |  |
| SAPS II score | 48 [37 ; 62] | 43 [32 ; 61] | 38.5 [30 ; 52] | 37 [28 ; 48] | <0.01^c^ |
| SOFA score | 7 [5 ; 9] | 8 [6 ; 9] | 7 [5 ; 9] | 7 [5 ; 9] | 0.60 |
| SOFA respiratory item (>2) | 25 (42.37) | 15 (65.22) | 190 (64.63) | 160 (76.56) | <0.01^bc^ |
| SOFA cardio-vascular item (>2) | 8 (13.56) | 6 (26.09) | 144 (48.98) | 91 (43.54) | . |
| SOFA Kidney item (>2) | 17 (28.81) | 1 (4.35) | 61 (20.75) | 31 (14.83) | 0.02^a^ |
| Severity of ARDS |  |  |  |  |  |
| PaO2/FiO2 | 130.77 [94.18 ; 200] | 125 [74 ; 198.68] | 102 [69.41 ; 168] | 93 [66 ; 143] | <0.01^bc^ |
| Leucocytes on admission (G/L) | 9.6 [7.0 ; 13.84] | 9.0[6.37 ; 13.0] | 11.3[5.17 ; 17.8] | 9.4[4.1 ; 17.2] | 0.07 |
| Ventilatory support on admission |  |  |  |  |  |
| Mechanical ventilation on admission | 54 (91.53) | 19 (82.61) | 187 (63.61) | 133 (63.94) | <0.01^c^ |
| High flow nasal cannula | 4 (6.78) | 3 (13.04) | 102 (34.69) | 70 (33.65) | <0.01^cd^ |
| Continuous positive airway pressure | 8 (13.56) | 3 (13.04) | 34 (11.56) | 20 (9.62) | 0.81 |
| ECMO | 2 (3.39) | 0 | 9 (3.06) | 7 (3.37) | 0.85 |
| Paralytic agent | 37 (62.71) | 11 (47.83) | 169 (57.48) | 128 (61.54) | 0.50 |
| Prone position | 7 (11.86) | 1 (4.35) | 50 (17.01) | 65 (31.25) | <0.01^bd^ |
| Nitric oxide | 4 (6.78) | 2 (8.7) | 13 (4.42) | 14 (6.73) | 0.61 |
| Renal replacement therapy | 7 (11.86) | 0 | 24 (8.16) | 4 (1.92) | <0.01^b^ |
| Vasopressors | 11 (18.64) | 5 (21.74) | 131 (44.56) | 76 (36.54) | <0.01^c^ |
| Steroids | 18 (30.51) | 7 (30.43) | 141 (47.96) | 121 (57.89) | <0.01^bcd^ |
| Proton Pomp Inhibitors | 41 (69.49) | 15 (65.22) | 149 (50.68) | 112 (53.85) | 0.04^c^ |
| Any ATB treatments on admission | 31 (52.54) | 17 (73.91) | 232 (78.91) | 144 (68.9) | . |
| Amoxicillin | 2 (3.39) | 0 | 4 (1.36) | 2 (0.96) | 0.50 |
| Amoxicillin and clavulanic acid | 6 (10.17) | 6 (26.09) | 19 (6.46) | 16 (7.69) | <0.01^d^ |
| Ureido-carboxypenicillins | 13 (22.03) | 6 (26.09) | 24 (8.16) | 10 (4.81) | <0.01^cd^ |
| 3rd generation cephalosporins | 19 (32.2) | 9 (39.13) | 160 (54.42) | 110 (52.88) | 0.01^c^ |
| 4th generation cephalosporins | 2 (3.39) | 0 | 19 (6.46) | 12 (5.77) | 0.51 |
| Penems | 1 (1.69) | 0 | 10 (3.4) | 3 (1.44) | 0.43 |
| Macrolides | 20 (33.9) | 7 (30.43) | 101 (34.35) | 78 (37.5) | 0.84 |
| Aminoglycosides | 8 (13.56) | 2 (8.7) | 30 (10.2) | 11 (5.29) | 0.13 |
| Fluoroquinolones | 4 (6.78) | 2 (8.7) | 23 (7.82) | 9 (4.33) | 0.45 |
| Anti-MSSA* | 3 (5.08) | 0 | 5 (1.7) | 0 | 0.02 |
| Anti MRSA** | 5 (8.47) | 1 (4.35) | 9 (3.06) | 3 (1.44) | 0.05^c^ |
| Lopinavir-Ritonavir | 0 | 0 | 55 (18.71) | 44 (21.15) | <0.01^cd^ |
| Hydroxychloroquine | 0 | 0 | 19 (6.46) | 15 (7.21) | 0.11 |
| Remdesivir | 0 | 0 | 21 (7.14) | 24 (11.48) | 0.01^c^ |
| Ozeltamivir | 21 (35.59) | 9 (39.13) | 14 (4.76) | 8 (3.85) | <0.01^cd^ |
| Co-infections |  |  |  |  |  |
| Bacterial pneumonia | 18 (30.51) | 9 (39.13) | 34 (11.56) | 22 (10.53) | <0.01^cd^ |
| Characteristics before intubation |  |  |  |  |  |
| Leucocytes before intubation (G/L) | 11.0[7.66 ; 16.1] | 11.2[8.0 ; 16.0] | 11.8[5.17 ; 17.65] | 9.2[4.49 ; 17.3] | 0.41 |
| PaO2/FiO2 | 133.3 [102 ; 203.3] | 116.7 [74 ; 174] | 116.7 [82.5 ; 172] | 115 [75 ; 148] | 0.02^b^ |
| Compliance (missing=230) | 27.5 [22.8 ; 31.4] | 25.8 [19.4 ; 31.3] | 28.2 [22.2 ; 36.9] | 29.1 [20.9 ; 37.8] | 0.70 |
| Peep (missing=49) | 10 [8 ; 12] | 8 [6.5 ; 10] | 12 [10 ; 13] | 12 [10 ; 14] | <0.01^bc^ |
| Peep > 12 mmHg | 6 (10.2) | 1 (4.3) | 74 (25.2) | 76 (36.4) | <0.01^bcd^ |
| Prone position | 8 (13.6) | 3 (13) | 81 (27.6) | 86 (41.1) | <0.01^bcd^ |
| Blocking agent | 37 (62.7) | 14 (60.9) | 233 (79.3) | 172 (82.3) | <0.01^cd^ |
| SDD§ | - | - | 26 (8.8) | 28 (13.4) | - |
| Treatments during ICU stay |  |  |  |  |  |
| Prone position | 12 (20.34) | 6 (26.09) | 116 (39.46) | 146 (69.86) | <0.01^bcd^ |
| Nitric oxide | 4 (6.78) | 7 (30.43) | 69 (23.47) | 77 (36.84) | <0.01^d^ |
| ECMO | 4 (6.78) | 1 (4.35) | 21 (7.14) | 33 (15.79) | <0.01^d^ |
| Renal replacement therapy | 21 (35.59) | 3 (13.04) | 87 (29.59) | 74 (35.41) | 0.11 |
| Vasopressors | 11 (18.64) | 8 (34.78) | 204 (69.39) | 137 (65.55) | <0.01^cd^ |
| ICU LOS | 13 [7 ; 20] | 27 [19 ; 45] | 13 [8 ; 19] | 25 [16 ; 38] | <0.01^ab^ |
| Hospital LOS | 21 [13 ; 41] | 46 [35 ; 57] | 17 [11 ; 30] | 31 [19 ; 50] | <0.01^abc^ |
| Day 60 death | 15 (25.42) | 4 (17.39) | 134 (45.58) | 99 (47.37) | <0.01^cd^ |

Inf: influenza; VAP: ventilator associated pneumonia; COV: COVID-19; ICU: intensive care unit; SAPS II: simplified acute physiology score (SAPS) I; SOFA: sequential organ failure assessment; ARDS: acute respiratory distress failure; ECMO: extra corporeal membrane oxygenation; ATB: antimicrobial; MSSA: methicillin susceptible staphylococcus aureus; MRSA: methicillin resistant staphylococcus aureus; LOS: length of stay; SDD: selective digestive contamination

* Cefazoline – Peni M ; ** Daptomycin – Linezolide -Vancomycine

§Without intravenous antimicrobial therapy

Comparisons with a p-value < 0.05: a, between VAP and non-VAP in influenza patients; b, between VAP and non-VAP in COVID patients; c, between influenza and covid patients in patients without VAP; d, between influenza and COVID patients **in patients**? with VAP

Table S 3: Risk factors of day-60 mortality in the whole cohort - Univariate analyses – Survival Cox models

| Variables (Median [IQR]/n(%)) | Alive | Dead | HR | CI 95% HR | p-value |
| --- | --- | --- | --- | --- | --- |
| Number of patients | 333 | 252 |  |  |  |
| Period of admission |  |  |  |  |  |
| Before 1January, 2020 | 63 (18.9) | 19 (7.5) | 0.56 | [0.31; 0.98] | 0.04 |
| From 1August, 2020 to 31 December, 2020 | 44 (13.2) | 57 (22.6) | 1.40 | [0.97; 2.01] | 0.07 |
| From 1 January, 2021 | 60 (18) | 64 (25.4) | 1.22 | [0.86; 1.73] | 0.27 |
| From 1January, 2020, to 31July, 2020 | 166 (49.8) | 112 (44.4) | 1 |  | 0.02 |
| Age >70 yo | 80 (24) | 112 (44.4) | 4.01 | [2.38 ; 6.75] | <0.01 |
| Between 60 and 70 yo | 84 (25.2) | 84 (33.3) | 3.10 | [1.83 ; 5.25] | <0.01 |
| Between 50 and 60 yo | 98 (29.4) | 39 (15.5) | 1.45 | [0.82 ; 2.56] | 0.21 |
| < 50 yo | 71 (21.3) | 17 (6.7) |  |  |  |
| Male gender | 232 (69.7) | 183 (72.6) | 1.09 | [0.82 ; 1.44] | 0.56 |
| Body-mass index ≥ 30 kg/m² | 125 (37.5) | 100 (39.7) | 1.04 | [0.8 ; 1.34] | 0.77 |
| Comorbidities | 200 (60.1) | 196 (77.8) |  |  |  |
| Immunodeficiency | 30 (9) | 48 (19) | 1.94 | [1.41 ; 2.69] | <0.01 |
| Time between symptoms and ICU admission > 10 days | 99 (29.7) | 59 (23.4) | 0.72 | [0.54 ; 0.97] | 0.03 |
| Time between ICU admission and OTI > 5 days | 18 (5.4) | 52 (20.6) | 2.59 | [1.85 ; 3.62] | <0.01 |
| Pneumonia on admission | 50 (15) | 33 (13.1) | 0.89 | [0.61 ; 1.29] | 0.54 |
| MDR colonization on admission | 16 (4.8) | 20 (7.9) | 1.20 | [0.76 ; 1.92] | 0.43 |
| Treatements on admission |  |  |  |  |  |
| Corticosteroids | 141 (42.3) | 146 (57.9) | 1.35 | [1.02 ; 1.8] | 0.04 |
| Anti-inflammatory (Anakinra/ Tocilizumab) | 38 (11.4) | 39 (15.5) | 1.13 | [0.79 ; 1.61] | 0.51 |
| Remdesivir | 17 (5.1) | 28 (11.1) | 1.48 | [0.99 ; 2.23] | 0.06 |
| Antimicrobial therapy | 238 (71.5) | 172 (68.3) | 0.88 | [0.67 ; 1.16] | 0.36 |
| % time ATB before intubation >=50% | 90 (27) | 99 (39.3) | 1.45 | [1.12 ; 1.88] | <0.01 |
| Broad spectrum ATB | 37 (11.1) | 47 (18.7) | 1.57 | [1.14 ; 2.17] | 0.01 |
| Penems | 8 (2.4) | 8 (3.2) | 1.15 | [0.57 ; 2.33] | 0.70 |
| During the first 2 days of intubation |  |  |  |  |  |
| SOFA coagulation (>2) | 7 (2.1) | 11 (4.4) | 2.25 | [1.21 ; 4.17] | 0.01 |
| SOFA cardio-vascular (>2) | 166 (49.8) | 142 (56.3) | 1.29 | [0.94 ; 1.78] | 0.12 |
| SOFA Neurology (>2) | 86 (25.8) | 65 (25.8) | 1.00 | [0.71 ; 1.39] | 0.99 |
| SOFA Liver (>2) | 3 (0.9) | 2 (0.8) | 0.95 | [0.23 ; 3.85] | 0.94 |
| SOFA renal (>2) | 32 (9.6) | 62 (24.6) | 2.03 | [1.51 ; 2.73] | <0.01 |
| SOFA respiratory (>2) | 274 (82.3) | 214 (84.9) | 1.01 | [0.71 ; 1.44] | 0.96 |
| Peep > 12 cmH_2_O | 76 (22.8) | 81 (32.1) | 1.27 | [0.97 ; 1.67] | 0.08 |
| Prone position | 102 (30.6) | 76 (30.2) | 1.19 | [0.89 ; 1.59] | 0.24 |
| Paralytic agents | 254 (76.3) | 202 (80.2) | 1.34 | [0.97 ; 1.85] | 0.07 |
| ECMO | 11 (3.3) | 14 (5.6) | 1.22 | [0.69 ; 2.13] | 0.49 |
| RRT | 19 (5.7) | 44 (17.5) | 2.12 | [1.52 ; 2.96] | <0.01 |
| Vasopressors | 166 (49.8) | 142 (56.3) | 1.29 | [0.94 ; 1.78] | 0.12 |
| Enteral feeding | 208 (62.5) | 145 (57.5) | 0.88 | [0.65 ; 1.19] | 0.39 |
| Parenteral feeding | 41 (12.3) | 65 (25.8) | 1.71 | [1.17 ; 2.5] | 0.01 |
| Proton pump inhibitors | 202 (60.7) | 148 (58.7) | 1.05 | [0.79 ; 1.4] | 0.74 |
| Broad spectrum ATB | 55 (16.5) | 77 (30.6) | 1.66 | [1.26 ; 2.2] | <0.01 |
| Penems | 14 (4.2) | 14 (5.6) | 1.09 | [0.63 ; 1.87] | 0.76 |
| Selective digestive decontamination* | 23 (6.9) | 31 (12.3) | 1.17 | [0.78 ; 1.74] | 0.44 |
|  |  |  |  |  |  |
| SARS-CoV2 pneumonia | 270 (81.1) | 233 (92.5) | 2.03 | [1.17 ; 3.53] | 0.01 |
| VAP | 129 (38.7) | 103 (40.9) | 1.63 | [1.24 ; 2.13] | <0.01 |
| Adequate AT and VAP | 75 (22.5) | 62 (24.6) | 1.71 | [1.25 ; 2.33] | <0.01 |
| Inadequate AT and VAP | 81 (24.3) | 56 (22.2) | 1.19 | [0.86 ; 1.64] | 0.29 |
| Early VAP | 43 (12.9) | 48 (19) | 1.33 | [0.96 ; 1.84] | 0.09 |
| Late VAP without early VAP | 86 (25.8) | 55 (21.8) | 1.56 | [1.13 ; 2.15] | 0.01 |

yo: years old; ICU: intensive care unit; OTI: Oro tracheal intubation; MDR: multi drug resistant; ATB: antimicrobial therapy; SOFA: sequential organ failure assessment; Peep: positive end expiratory pressure; ECMO: extra corporeal membrane oxygenation; RRT: renal replacement therapy; HR: hazard ratio; CI: confidence interval; AT: antimicrobial therapy.

*without intravenous antimicrobial therapy

Table S 4: Association between VAP and day-60 mortality - multivariate survival Cox models

|  | aHR | CI 95% HR | p-value |
| --- | --- | --- | --- |
| Influenza (N=87) * | | | |
| VAP | 1.75 | [0.48; 6.33] | 0.36 |
| Adequate AT and VAP | 0.82 | [0.10; 6.89] | 0.85 |
| Inadequate AT and VAP | 2.19 | [0.58; 8.35] | 0.25 |
| Early VAP | 5.18 | [0.93; 29.00] | 0.06 |
| Late VAP# | 0.85 | [0.18; 4.11] | 0.84 |
| COVID-19 (N=499) ** | | | |
| VAP | 1.76 | [1.33; 2.34] | <0.01 |
| Adequate AT and VAP | 1.67 | [1.21; 2.30] | <0.01 |
| Inadequate AT and VAP | 1.34 | [0.95; 1.88] | 0.09 |
| Early VAP | 1.34 | [0.95; 1.88] | 0.09 |
| Late VAP# | 1.76 | [1.25; 2.47] | <0.01 |
| COVID-19 and Influenza (N=586) *** § | | | |
| VAP | 1.80 | [1.37; 2.36] | <0.01 |
| COVID-19 | 2.33 | [1.37; 3.95] | <0.01 |
| Adequate AT and VAP | 1.64 | [1.19; 2.24] | <0.01 |
| COVID-19 | 2.37 | [1.39; 4.03] | <0.01 |
| Inadequate AT and VAP | 1.45 | [1.05; 2.02] | 0.03 |
| COVID-19 | 2.44 | [1.43; 4.14] | <0.01 |
| Early VAP | 1.45 | [1.05; 2.02] | 0.03 |
| COVID-19 | 2.37 | [1.40; 4.03] | <0.01 |
| Late VAP# | 1.68 | [1.21; 2.33] | <0.01 |
| COVID-19 | 2.47 | [1.45; 4.20] | <0.01 |

AT: antimicrobial therapy; VAP: Ventilator Associated Pneumonia

*Adjustment for age, immunosuppression, steroids on admission, renal SOFA before VAP

**Adjustment for age, comorbidities, immunosuppression, time from admission to intubation > 5 days, time from symptoms to ICU admission > 10 days, Broad spectrum antimicrobial therapy, Renal SOFA > 2, ECMO, parenteral feeding

***Adjustment for age, comorbidities, immunosuppression, time from admission to intubation > 5 days, time from symptoms to ICU admission > 10 days, Cardio SOFA > 2, Renal SOFA > 2, blocking agent, parenteral feeding,

§interactions were tested and there was no interaction between VAP and COVID-19; early VAP and COVID-19 and late VAP and COVID-19.

#Late VAP without early VAP

Figure S1: Directed Acyclic Graph - (A)Unadjusted and (B) adjusted

| **(A)** |
| --- |
| 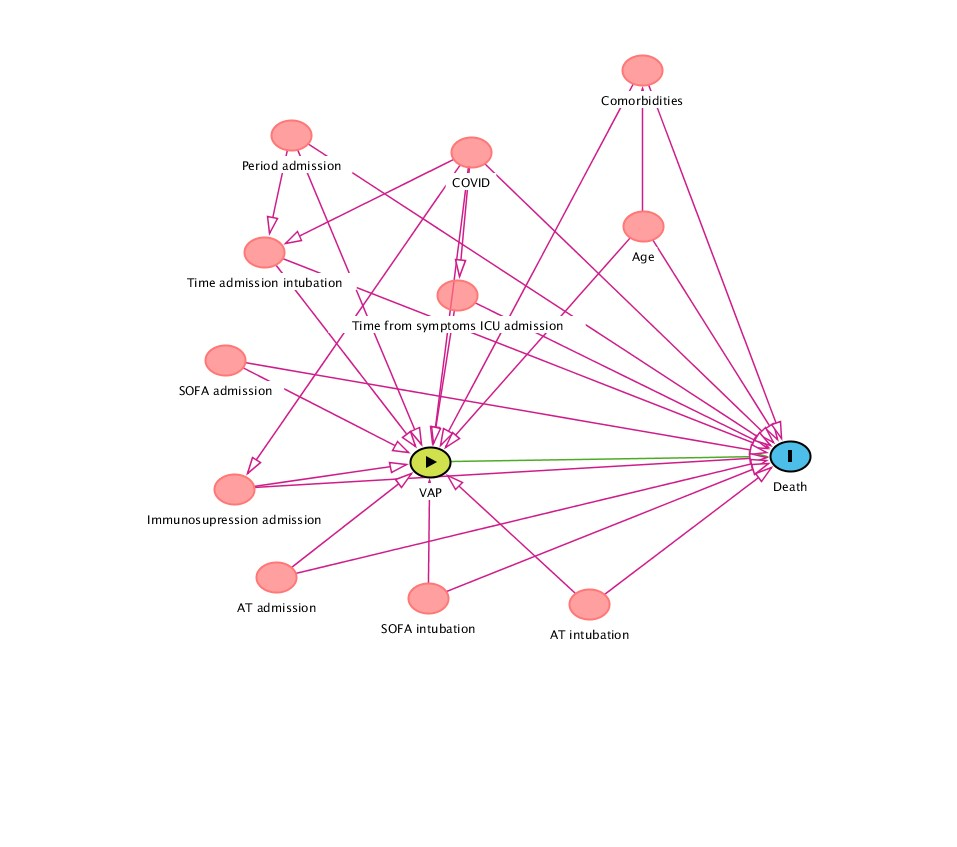 |
| **(B)** |
| 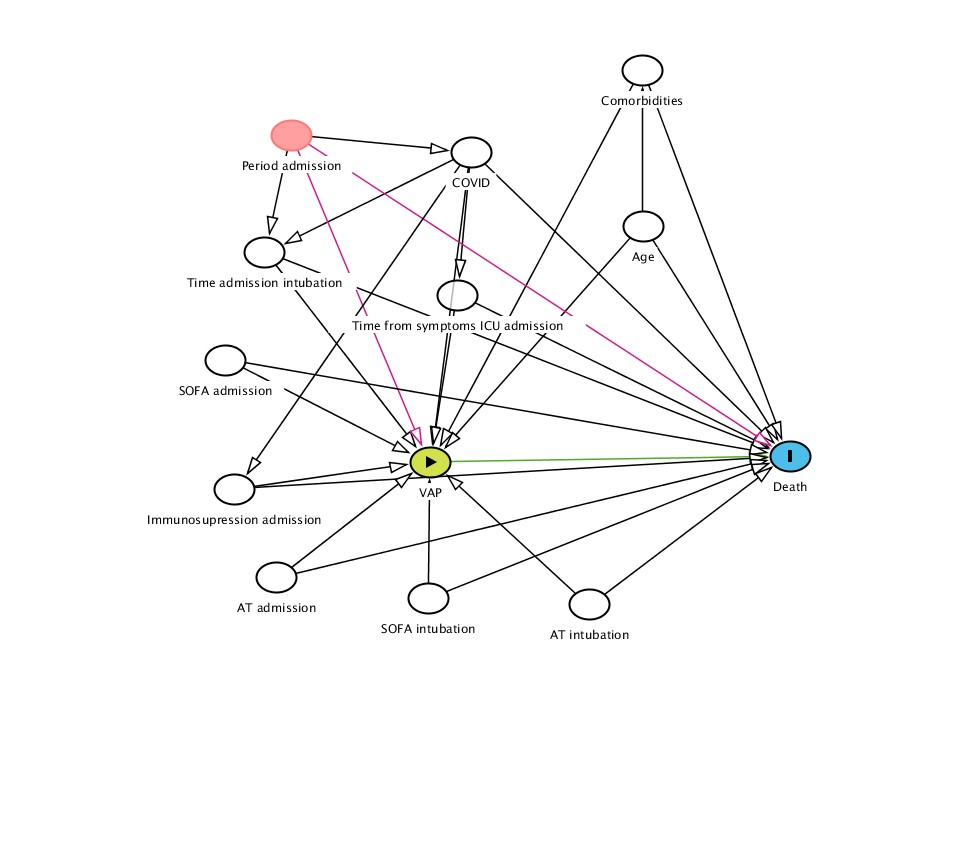 |
| “VAP” represents the exposure variable and “Death” the outcome variable. Biasing paths are shown in red and causal paths in green. |

| 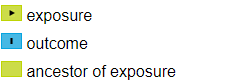 | 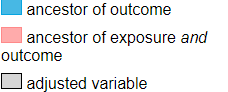 | 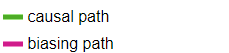 |
| --- | --- | --- |

Table S 5: Factors associated with day-60 death, multivariate cox model, section of the covariates with DAG

|  | COVID-19 and Influenza (N=586) | | | | | | | COVID-19 (N=499) | | | | | | | Influenza (N=87) | | | | | | |
| --- | --- | --- | --- | --- | --- | --- | --- | --- | --- | --- | --- | --- | --- | --- | --- | --- | --- | --- | --- | --- | --- |
| Parameter | HR | 95% CI | | | | | Pval | HR | 95% CI | | | | | Pval | HR | 95% CI | | | | | Pval |
| Time between symptoms and  ICU admission > 10 days | 0.78 | [ | 0.57 | ; | 1.05 | ] | 0.1 | 0.77 | [ | 0.57 | ; | 1.05 | ] | 0.1 |  |  |  |  |  |  |  |
| Age | 1.04 | [ | 1.02 | ; | 1.05 | ] | <0.01 | 1.04 | [ | 1.02 | ; | 1.05 | ] | <0.01 | 1.05 | [ | 1.01 | ; | 1.1 | ] | 0.02 |
| Comorbidities | 1.36 | [ | 0.99 | ; | 1.88 | ] | 0.06 | 1.36 | [ | 0.98 | ; | 1.89 | ] | 0.07 | 1.77 | [ | 0.21 | ; | 15.15 | ] | 0.6 |
| Immunodeficiency | 1.58 | [ | 1.09 | ; | 2.28 | ] | 0.02 | 1.43 | [ | 0.95 | ; | 2.15 | ] | 0.09 | 3.93 | [ | 1.28 | ; | 12.04 | ] | 0.02 |
| BMI > 30 kg/m² | 1.06 | [ | 0.81 | ; | 1.39 | ] | 0.68 | 1.01 | [ | 0.76 | ; | 1.34 | ] | 0.94 | 1.85 | [ | 0.65 | ; | 5.32 | ] | 0.25 |
| BMR colonization | 1.05 | [ | 0.65 | ; | 1.7 | ] | 0.85 | 1.02 | [ | 0.62 | ; | 1.7 | ] | 0.93 | 1.4 | [ | 0.27 | ; | 7.27 | ] | 0.69 |
| COVID | 2.11 | [ | 1.19 | ; | 3.75 | ] | 0.01 |  |  |  |  |  |  |  |  |  |  |  |  |  |  |
| Steroids on admission | 0.95 | [ | 0.7 | ; | 1.29 | ] | 0.76 | 0.99 | [ | 0.71 | ; | 1.38 | ] | 0.97 | 0.3 | [ | 0.08 | ; | 1.18 | ] | 0.09 |
| Other immunomodulatory  treatment on admission | 1.03 | [ | 0.7 | ; | 1.51 | ] | 0.89 | 1.07 | [ | 0.73 | ; | 1.58 | ] | 0.73 |  |  |  |  |  |  |  |
| Remdesivir | 1.1 | [ | 0.72 | ; | 1.69 | ] | 0.67 | 1.08 | [ | 0.7 | ; | 1.67 | ] | 0.72 |  |  |  |  |  |  |  |
| Antibacterial therapy on admission | 0.99 | [ | 0.74 | ; | 1.33 | ] | 0.93 | 1.04 | [ | 0.76 | ; | 1.42 | ] | 0.83 | 0.58 | [ | 0.21 | ; | 1.6 | ] | 0.29 |
| Pneumonia on admission | 1.08 | [ | 0.72 | ; | 1.6 | ] | 0.72 | 1.23 | [ | 0.81 | ; | 1.87 | ] | 0.34 | 0.83 | [ | 0.25 | ; | 2.7 | ] | 0.75 |
| SOFA on admission | 1.03 | [ | 0.97 | ; | 1.09 | ] | 0.36 | 1.03 | [ | 0.97 | ; | 1.09 | ] | 0.33 | 0.86 | [ | 0.53 | ; | 1.39 | ] | 0.54 |
| Intubation after day 5 | 2.43 | [ | 1.62 | ; | 3.65 | ] | <0.01 | 2.54 | [ | 1.66 | ; | 3.88 | ] | <0.01 | 0.6 | [ | 0.06 | ; | 5.82 | ] | 0.66 |
| SOFA on intubation | 1.08 | [ | 1.02 | ; | 1.15 | ] | 0.01 | 1.08 | [ | 1.02 | ; | 1.15 | ] | 0.01 | 1.28 | [ | 0.76 | ; | 2.16 | ] | 0.35 |
| VAP | 1.83 | [ | 1.38 | ; | 2.42 | ] | <0.01 | 1.87 | [ | 1.4 | ; | 2.51 | ] | <0.01 | 1.33 | [ | 0.37 | ; | 4.8 | ] | 0.67 |
